# GRETTA: an R package for mapping *in silico* genetic interaction and essentiality networks

**DOI:** 10.1093/bioinformatics/btad381

**Published:** 2023-06-16

**Authors:** Yuka Takemon, Marco A Marra

**Affiliations:** Genome Science and Technology Graduate Program, University of British Columbia, Vancouver, BC V5Z 4S6, Canada; Michael Smith Laboratories, University of British Columbia, Vancouver, BC V6T 1Z4, Canada; Canada’s Michael Smith Genome Sciences Centre, BC Cancer Research Institute, Vancouver, BC V5Z 4S6, Canada; Michael Smith Laboratories, University of British Columbia, Vancouver, BC V6T 1Z4, Canada; Canada’s Michael Smith Genome Sciences Centre, BC Cancer Research Institute, Vancouver, BC V5Z 4S6, Canada; Department of Medical Genetics, University of British Columbia, Vancouver, BC V6H 3N1, Canada

## Abstract

**Summary:**

Mapping genetic interactions and essentiality networks in human cell lines has been used to identify vulnerabilities of cells carrying specific genetic alterations and to associate novel functions to genes, respectively. *In vitro* and *in vivo* genetic screens to decipher these networks are resource-intensive, limiting the throughput of samples that can be analyzed. In this application note, we provide an R package we call Genetic inteRaction and EssenTiality neTwork mApper (GRETTA). GRETTA is an accessible tool for *in silico* genetic interaction screens and essentiality network analyses using publicly available data, requiring only basic R programming knowledge.

**Availability and implementation:**

The R package, GRETTA, is licensed under GNU General Public License v3.0 and freely available at https://github.com/ytakemon/GRETTA and https://doi.org/10.5281/zenodo.6940757, with documentation and tutorial. A Singularity container is also available at https://cloud.sylabs.io/library/ytakemon/gretta/gretta.

## 1 Introduction

Genetic perturbation screens, such as CRISPR-Cas9 knockout (KO) screens, have been used to uncover lethal and alleviating genetic interactions (GIs; Shalem *et al.* 2014, Hart *et al.* 2015, Wang *et al.* 2017, Behan *et al.* 2019) and essentiality networks to decipher a gene’s functional membership (Pan *et al.* 2018, Wainberg *et al.* 2021). Two genes are said to share a lethal, or synthetic lethal (SL), GI when the perturbation of both genes together in the same cell impedes cell viability, but perturbation of either gene individually has no impact on viability (Mani *et al.* 2008). Conversely, an alleviating GI occurs when the perturbation of two genes leads to a fitness advantage over cells with either gene perturbed individually (Mani *et al.* 2008). Lethal GIs of genes mutated in cancers have revealed tumor-specific vulnerabilities that can be therapeutically exploited. Most notable are the lethal GIs between *BRCA1/2* and *PARP1* (Bryant *et al.* 2005, Farmer *et al.* 2005), which led to the use of PARP inhibitors in BRCA1/2-deficient breast (Robson *et al.* 2017, 2019), ovarian (Mirza *et al.* 2018), and prostate cancers (Ramakrishnan Geethakumari *et al.* 2017). Essentiality network maps have been utilized to assign novel functions to genes (Wang *et al.* 2017, Kim *et al.* 2019, Wainberg *et al.* 2021, Pan *et al.* 2018). Two genes share co-essentiality when perturbation of either gene leads to similar fitness effects in cells. This is interpreted to indicate that the genes may have similar or synergistic functions (Kim *et al.* 2019, Wainberg *et al.* 2021). In contrast, two genes are antiessential when perturbations in either of them lead to opposite cellular fitness effects. This is interpreted to indicate genes with inhibitory or antagonistic relationships (Kim *et al.* 2019, Wainberg *et al.* 2021). It seems clear that mapping GI and essentiality networks of cancer-associated genes could reveal biological functions that might be leveraged to inform potential therapeutic strategies.

The Cancer Dependency Map (DepMap) public data platform (Meyers *et al.* 2017, Ghandi *et al.* 2019) is the largest resource for mining GIs and essentiality networks of human cancer cell lines. DepMap contains data from multi-omic characterization of cancer cell lines, including whole-genome sequencing, exome sequencing, RNA sequencing (RNA-seq) (Ghandi *et al.* 2019), proteomic quantification (Nusinow *et al.* 2020), and genome-wide CRISPR-Cas9 KO screens (Meyers *et al.* 2017), which are updated bi-annually with additional cancer cell lines (detailed in https://depmap.org/portal/announcements/). Novel algorithms utilizing DepMap data to predict GIs have emerged (De Kegel *et al.* 2021, Chiu *et al.* 2021, Benfatto *et al.* 2021). However, these tools either lack flexibility, the ability to detect alleviating GIs, or require advanced programming knowledge. Thus, for various reasons, these tools have limited accessibility to the larger research community.

To facilitate discoveries using GI and essentiality network mapping, we created an R package called Genetic inteRaction and EssenTiality neTwork mApper (GRETTA), which leverages the DepMap data platform. GRETTA provides a simplified approach to query DepMap for user-defined cell line features, including selection by gene, mutation, and cancer types, to predict both lethal and alleviating GIs and essentiality networks. We provide an intuitive method for visualizing results that require only a basic understanding of the R programming language. In addition to the case study, we provide here, we have used GRETTA to reveal functional networks of CIC, a cancer-associated gene (Takemon *et al.* 2023). We have thus demonstrated the utility of our tool for discovering genetic interaction and essentiality networks.

## 2 Materials and methods

### 2.1 Performing *in silico* GI screens

**Figure 1:**
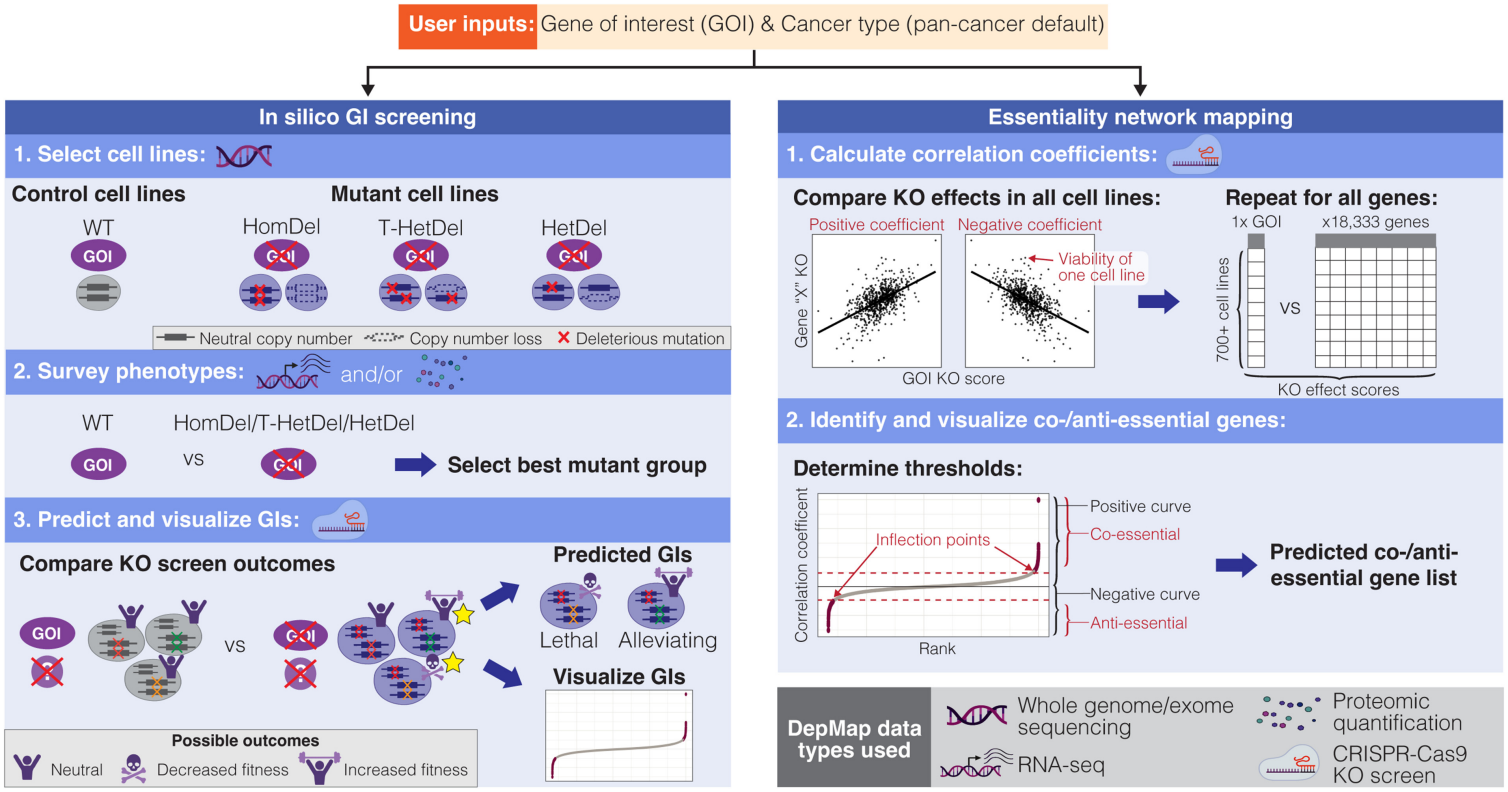
An overview of *in silico* GI screening and essentiality mapping using GRETTA. Top panel: A user defines a GOI and/or a cancer type to map GI or essentiality networks. When a cancer type is not defined, GRETTA defaults to a pan-cancer GI screen or essentiality mapping. Left panel: A three-step workflow to predict and visualize candidate GIs of a user-defined GOI in the defined cancer type. After cell lines are grouped into control or mutant groups, the user must select one mutant group based on gene and protein expression phenotypes. The genome-wide KO screen data for this user-selected mutant cell line group are then compared to those of the control group to predict lethal and alleviating GIs. Right panel: Pearson correlation analyses are performed between the KO effect scores of the GOI and each gene that was targeted in DepMap’s genome-wide screen (18 333 genes). The thresholds used to determine co-/antiessential genes are partly determined using inflection points of the ranked positive and negative coefficient curves. A detailed description of the workflow is provided in Supplementary Methods. The DepMap data type processed at each step is illustrated in each subheading and noted in the legend. This figure was partly created with BioRender.com.

GRETTA leverages cell line annotations, mutation annotations, copy number quantifications, RNA-seq, proteomic quantifications, and genome-wide CRISPR-Cas9 KO screen data that were made available by the DepMap data platform (Supplementary Methods; Meyers *et al.* 2017, Ghandi *et al.* 2019, Nusinow *et al.* 2020) and predicts candidate GIs in three steps: (i) selecting control and mutant cancer cell lines; (ii) surveying mRNA and/or protein expression changes; and (iii) predicting and visualizing GIs (illustrated in Fig. 1 with a detailed version provided in Supplementary Fig. S1A). Briefly, the first step is driven by a user-defined feature, such as a gene of interest (GOI). By default, when a GOI is provided, all pan-cancer cell lines with wildtype (WT) alleles and neutral copy number of the GOI are assigned to the control group, and lines with loss-of-function alterations in the GOI are assigned to one of the mutant subgroups, including homozygous deletion (HomDel), trans-heterozygous deletion, and heterozygous deletion groups (see Supplementary Methods for details). Cell lines can also be selected by specific mutation, cancer type, or manual curation (Supplementary Methods).

The second step involves extracting DepMap mRNA and protein expression data (if available) and performing differential mRNA and protein expression analyses of the GOI between control cell lines and mutant cell lines (Supplementary Methods). A mutant group with a significant reduction in mRNA or protein expression compared to the control group by Welch’s *t*-test is consistent with a potential reduction of GOI functions, and thus a user may select such a mutant cell line group for downstream analysis.

Finally, candidate GIs are predicted by conducting differential lethality probability analyses, which identify a gene KO that results in opposing survival outcomes in the mutant cell lines and control cell lines. Mann–Whitney *U* tests are performed by extracting the lethality probabilities of each of the 18 333 genes that were targeted in the DepMap CRISPR-Cas9 KO screen and comparing the control and mutant cancer cell line groups. Optionally, at this step, a list of genes can be provided to perform a small-scale screen. GRETTA also generates an interaction score that is used to visualize candidate GIs and to rank them from most to least likely to be lethal or alleviating (Supplementary Methods).

### 2.2 Mapping essentiality networks

GRETTA adapted a previously published Pearson correlation-based method for mapping essentiality between genes (Wang *et al.* 2017, Pan *et al.* 2018, Kim *et al.* 2019; illustrated in Fig. 1, Supplementary Fig. S1A; see Supplementary Methods for detailed description). Briefly, the genome-wide CRISPR-Cas9 KO screen data from DepMap is used to calculate pan-cancer essentiality of a gene in the following steps. First, KO effect scores of GOIs (a user-defined input) are extracted from all screened cancer cell lines. Next, Pearson’s correlation coefficients and *P*-values are calculated between the KO effect scores of the GOIs and 18 333 genes screened in the dataset. Next, the inflection point of the ranked positive and negative coefficient score curves is calculated to determine a threshold to identify co-essential (positively correlated) and antiessential (negatively correlated) genes. Finally, a ranked correlation coefficient score is provided as an output and is used to visualize essentiality networks.

## 3 Case study: predicting pan-cancer GIs and co-essentiality networks of *ARID1A*

*ARID1A* is a member of the mammalian switch/sucrose non-fermentable (SWI/SNF) complex (Mashtalir *et al.* 2018) with a known SL GI to its paralog *ARID1B* (Helming *et al.* 2014). Rationalizing that this known SL interaction and complex association would serve as positive controls, we applied GRETTA to predict pan-cancer GIs and essentiality networks of *ARID1A*, expecting to replicate known interactors and co-essential genes, respectively. We identified an *ARID1A* WT control group and an *ARID1A* HomDel mutant group, consisting of 906 and 23 cell lines, respectively. The *ARID1A* HomDel mutant group showed significantly reduced *ARID1A* mRNA expression compared to the control group (Welch’s *t*-test *P* <.05; Supplementary Fig. S2A and B and Table S1A and B), consistent with potentially reduced ARID1A function. Using these two groups, we found that *ARID1B* was predicted to be the top lethal genetic interactor of *ARID1A* (Supplementary Fig. S2C and Table S1C), thus confirming the established SL GI between these two genes. Furthermore, a pan-cancer essentiality analysis of *ARID1A* revealed the expected top five list of co-essential genes that are known mSWI/SNF complex members, namely *ARID1A, SMARCB1*, *SMARCE1*, *SMARCC1*, and *SS18* (Mashtalir *et al.* 2018; Supplementary Fig. S2D and Table S1D). GRETTA was thus able to recapitulate known GIs and co-essential networks of *ARID1A*.

## 4 Discussion

GRETTA was built to provide an easy-to-use method for mapping GI and essentiality networks. The R package requires only a basic understanding of the R programming language and we provide an easy-to-follow tutorial (https://github.com/ytakemon/GRETTA). Using GRETTA, we replicated known GIs and essentiality networks of ARID1A, thus demonstrating how the tool can be used and ultimately applied to discover GI and essentiality networks of other GOI. We are currently aware of 365 visitors and 135 total unique installations of GRETTA and from this deduce that GRETTA is of interest to the wider research community.

## Supplementary Material

btad381_Supplementary_Data

## Data Availability

The GRETTA R package, data analyzed in this study (DepMap version 22Q2), and the code used to generate the supplementary figures and tables are publicly available as a tutorial provided on the GRETTA GitHub repository (https://github.com/ytakemon/GRETTA). GRETTA has been archived with a citable DOI on Zenodo (https://doi.org/10.5281/zenodo.6940757) and a Singularity container has been made available on Sylabs (https://cloud.sylabs.io/library/ytakemon/gretta/gretta) to ensure reproducibility. GRETTA is shared under the GNU General Public License v3.0.
