# Supplementary material for: GRETTA: an R package for mapping in silico genetic interaction and essentiality networks
Source: Bioinformatics. 2023 Jun 16;39(6):btad381. doi: 10.1093/bioinformatics/btad381 (PMC10284671; doi:10.1093/bioinformatics/btad381)
Supplement: btad381_Supplementary_Data [file btad381_supplementary_data.zip › Supplemental Figures and Tables.docx]

# Supplemental Figures

#

###
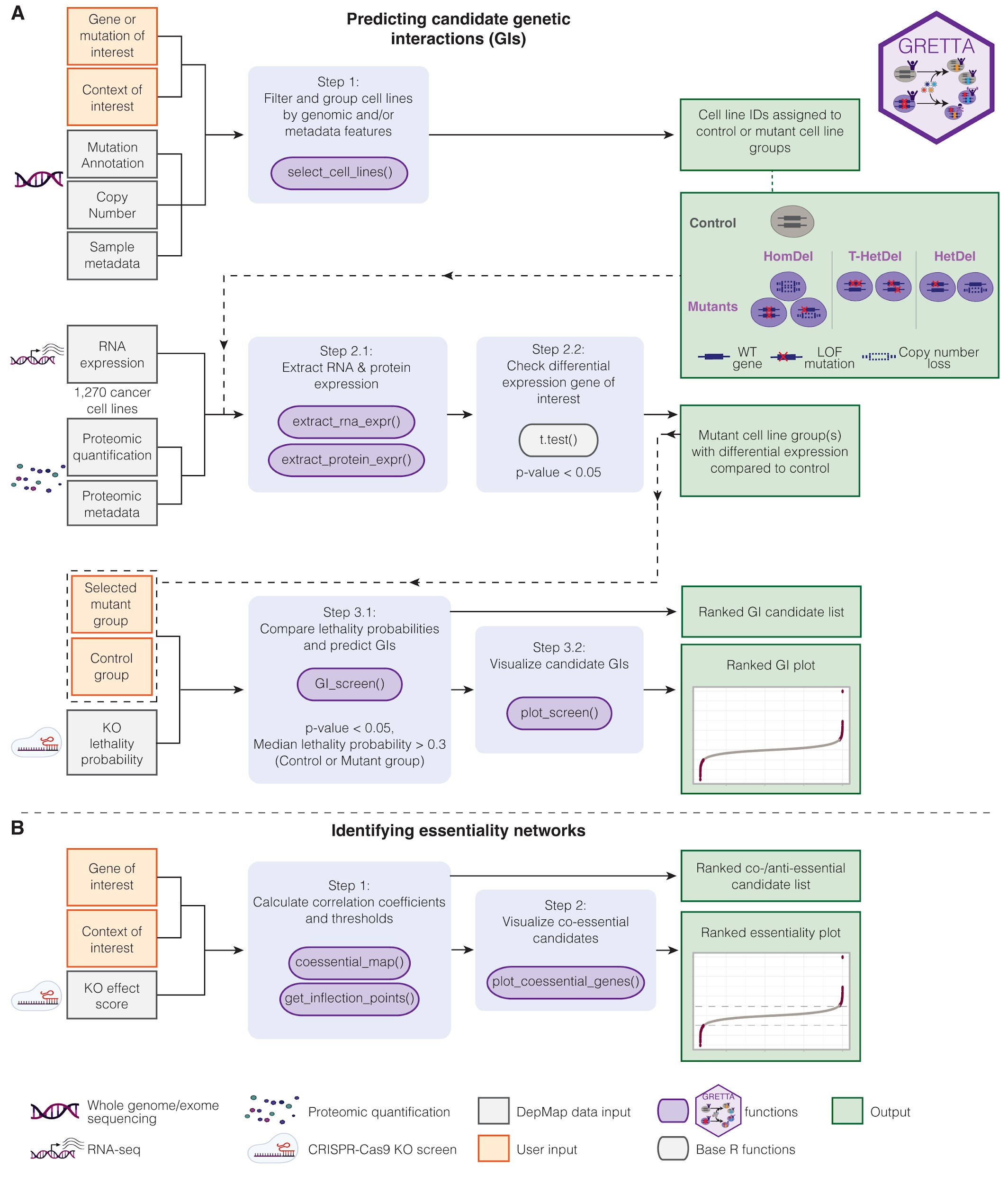


**Supplemental Figure S1.** A workflow of two GRETTA package analysis modes. A) A four-step workflow to predict and visualize candidate GIs of a user-defined gene and/or cell feature of interest. Final outputs consist of a comma-separated (.csv) file containing a ranked list of candidate genes that are potential GIs of the gene and/or cell feature of interest, and a ranked scatter plot with top lethal and alleviating GIs labeled for visualization. B) A two-step workflow to identify co-essential and anti-essential genes of a user-defined gene and/or cell feature of interest. Final outputs consist of a .csv file containing a ranked list of co-essential and anti-essential genes, and a ranked scatter plot to quickly visualize genes that are most likely to share essentiality. Icons from BioRender.com were incorporated in this figure.


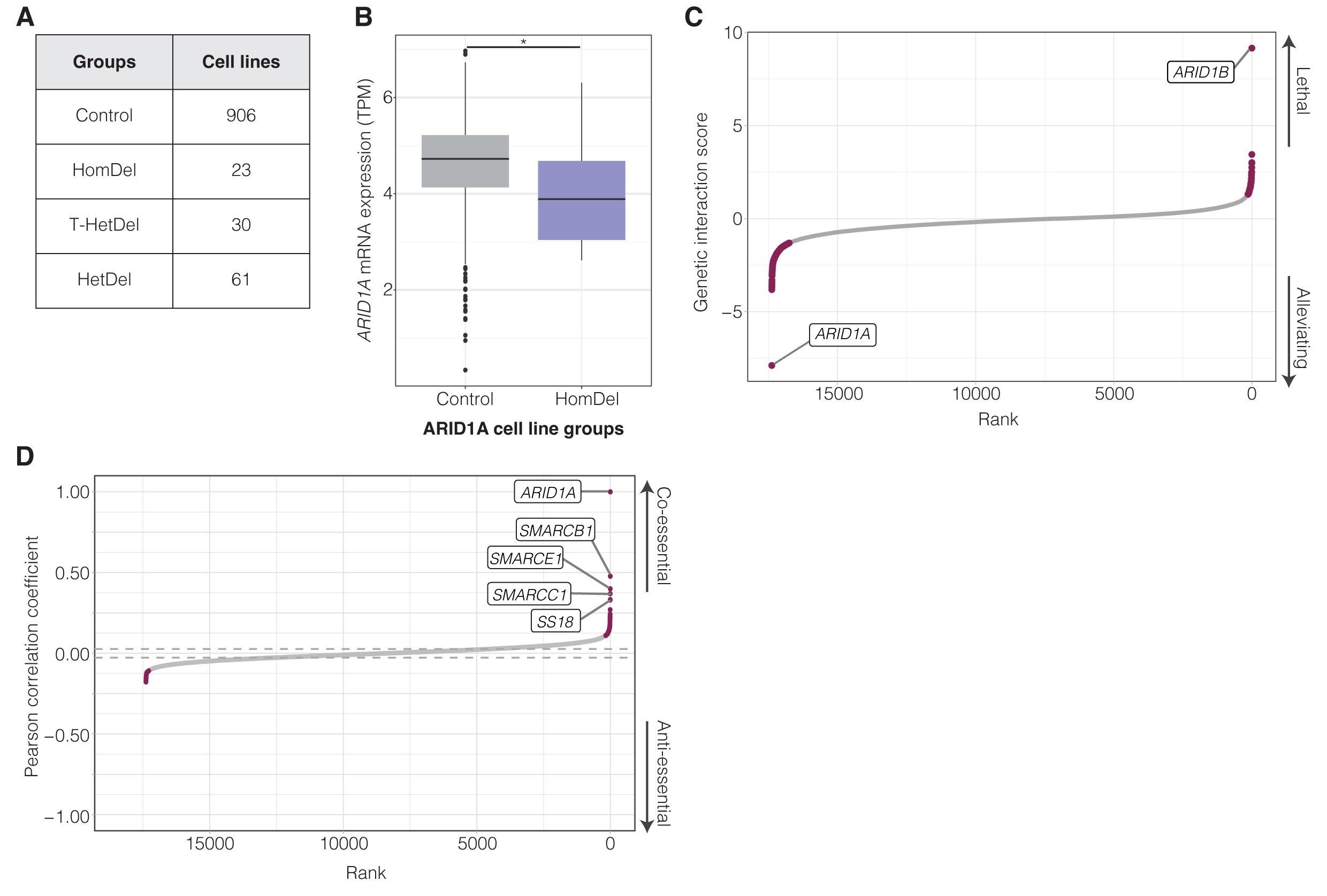


**Supplemental Figure S2.** Predicting *ARID1A* GIs and co-essential genes. A) Number of cancer cell lines in *ARID1A* control and LOF mutant groups identified using default pan-cancer settings. B) Tukey boxplots of *ARID1A* mRNA expression in *ARID1A* control (831 lines) and HomDel mutant groups (23 lines). Welch’s T-test p-value * < 0.05. C) Ranked *ARID1A* genetic interaction scores generated using GRETTA. Purple points indicate candidate *ARID1A* GIs (787 total candidate genes, of which 639 were alleviating interactors and 145 were lethal interactors; Mann-Whitney U-test p-value < 0.05). The top most lethal and alleviating genetic interactors are labeled. D) A ranked *ARID1A* coefficient plot showing candidate co-essential genes and anti-essential genes (purple points; BH-adjusted p-value < 0.05 and past inflection point of the positive or negative curve) generated using GRETTA. Dashed horizontal lines denote the inflection points of the positive and negative correlation curve. Top five co-essential genes are labeled.

# Supplemental Table Legend

**Supplemental Table S1.** GRETTA outputs of *ARID1A* *in silico* GI screen and essentiality mapping case study. A) Pan-cancer *ARID1A* control and mutant cell line groups were identified using the default settings of `select_cell_lines()`. B) *ARID1A* mRNA expression retrieved for *ARID1A* control (831 lines) and HomDel mutant groups (23 lines) using `extract_rna()`. C) Output of `GI_screen()` comparing *ARID1A* control and HomDel cancer cell lines showing candidate lethal and alleviating GIs of *ARID1A*. D) Output of `coessential_map()` showing *ARID1A*’s pan-cancer candidate co-essential and anti-essential genes.
